# Supplementary material for: What is the added value of incorporating pleasure in sexual health interventions? A systematic review and meta-analysis
Source: PLoS One. 2022 Feb 11;17(2):e0261034. doi: 10.1371/journal.pone.0261034 (PMC8836333; doi:10.1371/journal.pone.0261034)
Supplement: S1 Table — (DOCX) [file pone.0261034.s001.docx]

| Study | Design | Participant characteristics | *n* at baseline | Intervention | Pleasure component | Time to follow-up | Sexual health outcome |
| --- | --- | --- | --- | --- | --- | --- | --- |
| Andrade et al., (2009)*  Brazil  The PEAS Belgo Program | Quasiexperimental with pre- and post-tests and control group | Students attending school in grades 6-8 and between ages 10-19 | 2737 | Active participatory school based program, with activities throughout the year with health and educational professionals  Control: No intervention given | Focused on the positive aspects of sexuality, emphasized the importance of intra and interpersonal relationships in the context of a healthy and pleasurable sex life, and stimulated gender equity with the aim of reducing the vulnerability of adolescents. | 18 months | Ever having engaged in sexual practices:  OR = 0·82, 95% CI 0·61 – 1·10, p = 0·186  Consistent condom use with casual partner  OR = 0·61**, 95% CI 0·38 – 0·99, p = 0·046  Consistent condom use with steady partner  OR = 0·87, 95% CI 0·56 – 1·34, p = 0·522  Use of modern contraceptive at last intercourse  OR = 0·79, 95% CI 0·55 – 1·14, p = 0·208 |
| Bauermeister et al. (2019)*  USA  The myDEx Project | RCT | Young gay, and bisexual, and other men who have sex with men  Mean age 21.7 | 180 | Online intervention with 6 sessions involving brief activities and videos designed to build HIV risk reduction skills and promote self-reflection about sexual health and partner-seeking behaviors  Control: Attention control, HIV risk reduction information only site | Session 1: focus on importance of feeling comfortable talking about sexuality, desires within relationships, and health  Session 3: comprehensive sex education review focused on same-sex behaviors, including the importance of sex positivity, varying sexual practices, and sexual consent | 30 days  60 days  90 days | Engaging in condomless receptive anal sex (90days)  OR = 0·43**, 95% CI 0·20 – 0·94, p = 0·04  Engaging in condomless insertive anal sex (90days)  OR = 0·64, 95% CI 0·28 – 1·44, p = 0·27 |
| Brown et al. (2019)  USA | RCT | HIV-infected men who have sex with men  Mean age 40.6 | 80 | Two group sessions, each lasting four hours, promoting sexual health and stress management, workshops  Control: Delayed intervention | Session 2: Healthy Relationships which included:  Practice and know how to correctly use a condom while still making the experience enjoyable | 3 months | HIV transmission knowledge  $\eta^{2}$ = 0·22***, p < 0·001  Intentions to refuse unprotected sex  $\eta^{2}$ = 0·06*, p = 0·05  Unprotected anal sex  $\eta^{2}$ = 0·02, p = 0·20  Unprotected anal or oral sex  $\eta^{2}$ = 0·07**, p = 0·03 |
| Champion & Collins (2011)  USA  Project IMAGE | RCT | Ethnic minority adolescent women with a history of abuse and STI between 14-18 years | 559 | Physical examination, two workshops, 3-5 support group sessions, and 2+ individual counselling  Control: Enhanced clinical counselling | Session 2: Commitment to change which included: Strategies to reduce risk behaviour  Talk about condom use including application and use for pleasure | 6 months  12 months | Infection during 0-12 months post-intervention  OR = 0·035**, 95% CI 0·02 – 0·53, p = 0·016  Infection during 6-12 months post-intervention  OR = 0·021***, 95% CI 0·01 – 0·39, p = 0·009 |
| Coleman et al. (2009)  USA | RCT | Older HIV-positive African American men who have sex with men  Age range 50-71 | 60 | Four 120-minute interactive sessions targeting HIV risk reduction over 4-week period.  Control: Four 120-minute general health group discussions over 4-week period | Condom use activities were designed to make condom use pleasurable and fun | Immediately after intervention  3 months | Consistent condom use (3 months)  OR = 2·04, 95% CI 0·48 – 8·77, p = 0·34  Consistent condom use in inconsistent users at baseline (3 months)  OR = 5·18*, 95% CI 0·97 – 27·78, p = 0·054 |
| Copenhaver, Lee, Baldwin (2013)  USA  Community-Friendly  Health Recovery Program | RCT | High-risk drug users in treatment  Median age 33 | 304 | 4 x 50 minutes group sessions, addresses sex and drug-related HIV risks  Control: Time and contact matched support and orientation group for people entering the methadone maintenance program | The Negotiating harm reduction with partners component included eroticizing safer sexual practices | Immediately after intervention  3 months  6 months  12 months | Model including all post-intervention data points:  Knowledge**, p <0·05  Female condom skills***, p < 0·001  Male condom skills***, p < 0·001  Condom use, p>0·05 |
| Crosby et al. (2014)*  USA  Focus on the Future (adapted) | RCT | Black male youths attending STI clinics between 15-23 years old  Mean age 19.6 | 702 | Brief, sex-positive intervention delivered as part of STI clinical care, approximately 50 minutes  Control: Attention-equivalent control consisting of PowerPoint slides focused on male and female anatomy, basic STI knowledge and prevention information. | Intervention addressed barriers to condom use, including the perception that condoms reduce sexual pleasure | 2 months  6 months | Condom use (2 months)  OR = 1·39, 95% CI 0·90 – 2·13, p = 0·14  Condom use (6 months)  OR = 1·63**, 95% CI 1·07 – 2·49, p = 0·02 |
| Crosby et al. (2018)  USA  Focus on the Future (adapted) | RCT | Young Black men who have sex with men between 15-29 years of age | 600 | Single 1:1 session theory-based, sex-positive clinic based safer sex program and free selection of high-quality condoms and lubricants with educator explaining use  Control: Access to condoms and lubricants (no accompanying educator) | Addressed how condom use can enhance sexual experience and pleasure | 12 months | Reference group for all comparisons is HIV- controls  Condom use for insertive anal sex  HIV+: OR = 0·71, 95% CI 0·33 – 1·51, p = 0·37  HIV-: OR = 0·86, 95% CI 0·47 – 1·58, p = 0·63  Condom use for receptive anal sex  HIV+: OR = 1·32*, 95% CI 0·97 – 1·78, p = 0·08  HIV-: OR = 1·17, 95% CI 0·92 – 1·48, p = 0·20  Consistent condom use for insertive anal sex  HIV+: OR = 0·37***, 95% CI 0·24 – 0·57, p < 0·001  HIV-: OR = 1·43*, 95% CI 0·94 – 2·19, p = 0·10  Consistent condom use for receptive anal sex  HIV+: OR = 1·63***, 95% CI 1·23 – 2·17, p = 0·001  HIV-: OR = 1·94***, 95% CI 01·57 – 2·39, p < 0·001 |
| Crosby et al., (2009)  USA  Focus on the Future | RCT | Young heterosexual African American men newly diagnosed with a STD  Mean age 23.1 | 266 | Personalized, single-session intervention delivered by a lay health adviser, 45-50 mins, access to free lubricants and 12+ condoms from an array of brands and sizes + standard care  Control: Standard care (nurse delivered messages regarding condom use + up to 12 free condom of single available size and brand) | Advisor encouraged men to feel good about using condoms, to experience condoms as being compatible with sexual pleasure | 3 months,  6 months | Reinfection  OR = 0·32**, 95% CI 0·12 – 0·86, p = 0·02  Condom skills  OR = 3·21***, 95% CI 2·80 – 3·63, p < 0·01  Unprotected acts of sexual intercourse in past 3 months  OR = -13·4 95% CI -35·6 – 8·8, p = 0·23  Condom used at last act of sexual intercourse  OR = 2·20**, 95% CI 1·08 – 4·48, p = 0·03 |
| Cruess et al. (2018)  USA  HINTS | RCT | HIV-positive minority gay and bisexual men  Mean age 44.7 | 167 | Theoretically derived, online delivered HIV sexual risk reduction intervention providing information, motivation, behavioral skills  Control: Healthy Living condition, matched format, discussion on non-sexual health | Topics included how to make condom use more enjoyable during sex | 6 months | Condomless anal sex – all partners  IRR = 0·96, 95% CI 0·61 – 1·522, p = 0·87  Condomless anal sex – HIV-/unknown partners  IRR = 0·37***, 95% CI 0·21 – 0·67 p = 0·001  Condomless anal sex – HIV+  IRR = 2·14***, 95% CI 1·27 – 3·62, p = 0·004 |
| Diallo et al. (2010)*  USA  The Healthy Love Workshop | RCT | Black Women  Mean age 31 | 313 | Single session affirming, black-woman-centred, sex-positive HIV prevention intervention. Delivered to pre-existing groups of women (eg. friends, sororities), in settings of their choosing (non-clinical).  Control: HIV 101 single session, didactic, lecture-style | Eroticization of safer sex. Aims to create a safe space in which black women can connect with their sexuality in ways that are positive and self-loving. | 3 months  6 months | Condom use with any male partner (3 months)  OR = 2·40***, 95% CI 1·28 – 4·50, p < 0·01  Condom use with any male partner (6 months)  OR = 1·50, 95% CI 0·83 – 2·72, p > 0·05  Condom use with primary male partner (3 months)  OR = 2·87**, 95% CI 1·18 – 6·95, p < 0·05  Condom use with primary male partner (6 months)  OR = 1·69, 95% CI 0·90 – 3·18, p > 0·05 |
| El-Bassel et al., (2011)  USA | RCT | Heterosexual couples from low-income urban communities who use drugs  Mean age 36.5 | 564 | Couple-based (Arm 1) or individual-based (Arm 2) risk reduction for HIV and drug use, based on social cognitive theory. Seven structured 2-hour sessions, delivered weekly.  Control: Couple wellness promotion | Component 3: practicing technical condom use placement skills along with a broader repertoire of pleasurable safer sex activities and syringe disinfection skills  Facilitators were trained to validate the relationship's strengths of commitment, love, trust, and empower the dyad to enact protective behaviors | Immediately after intervention  6 months  12 months | Reference group for all comparisons is wellness promotion arm  Consistent condom use with study partner (6 months)  OR = 2·16***, 95% CI 1·29 – 3·61, p < 0·01  Consistent condom use with study partner (12 months)  OR = 1·14*, 95% CI 0·54 – 2·40, p > 0·05  Consistent condom use with any partner (6 months)  OR = 1·61**, 95% CI 1·01 – 2·55, p < 0·05  Consistent condom use with any partner (12 months)  OR = 0·91, 95% CI 0·45 – 1·81, p > 0·05 |
| Ferrer et al. (2011)*  USA | RCT | Young adults enrolled in traditional college | 199 | Sexual risk reduction intervention addressing social-cognitive (SC) factors (Arm 1) or social-cognitive intervention + emotional education (SCE) (Arm 2)  Control: Standard of care (materials presented by university) | SCE intervention: Videos demonstrated ways that condom  use could lead to eroticism (e.g., putting a condom on with  one’s mouth) | 3 months  6 months | SCE arm compared with control:  Condom use (3 months)  d = 0·49**, p = 0·02  Condom use (6 months)  d = 0·56***, p < 0·01  SCE arm compared with SC arm:  Condom use (3 months)  d = 0·08, p = 0·41  Condom use (6 months)  d = 0·38**, p = 0·04 |
| Garcia-Vazquez, Quinto, Agullo-Tomas (2019)*  Spain  Neither Ogres Nor Princesses | Quasiexperimental with pre- and post-tests and control group | Adolescents in school between ages 12-16 | 656 | Secondary school program, educating students for 4 years with trained teaching staff and external workshops  Control: No intervention given | Presents sexuality as a positive human value and source of pleasure, with a gender and empowering perspective. One thematic block specifically discusses pleasure and health (sexual practices, consent, prevention, etc.) | Immediately after intervention  2 years | Knowledge immediately after*, p = 0·053  Knowledge after 2 years**, p = 0·022  Condom use at first time immediately after**, p < 0·05  Condom use at first time 2 years, p > 0·05  Condom use at last time immediately after, p > 0·05  Condom use at last time 2 years, p > 0·05 |
| Goldberg et al. (2009)  USA | RCT | Incarcerated youth between 13-18 years | 391 | Six one-hour education sessions over a 3 week period. (Arm 1) Booster group attended same sessions plus a booster session at the end of 3 months (Arm 2)  Control: No intervention, offered condensed HIV education session after study completion | Session 3: Discusses pleasure and comfort (for both females & males) during condom application and use module. One part also addresses how condom use can be made easier and more fun. | 1 month  3 months  6 months | Male participants:  Prevention attitude**, p = 0·017  Risk behavior***, p < 0·001  Condom attitude**, p = 0·022  Safer behavior, p = 0·70  Female participants:  Prevention attitude, p = 0·14  Risk behavior, p = 0·15  Condom attitude, p = 0·82  Safer behavior, p = 0·66 |
| Gollub et al. (2010)  USA  Women Fighting Infection Together | RCT | Women with drug use history  Age range 21-56 | 189 | Four-session delivered weekly (and one reunion session delivered on month later), peer-led, interactive group intervention that targeted body knowledge and HIV/STI prevention, including a focus on women’s health (reproductive health screening, sexual violence, self-breast examination, STI signs, symptoms)  Control: Personalized HIV risk reduction counselling, testing, limited case management | Group Session 2 included discussions of the notion of a woman’s own desires and sexual pleasure | 2 months | Body knowledge***, p < 0·001  Knowledge about prevention methods**, p = 0·014  Male condom use***, p < 0·001  Female condom use***, p < 0·001 |
| Heeren et al. (2013)  South Africa  Wake Up | RCT | University students  Mean age 20.8 | 176 | HIV risk reduction intervention comprising eight 45-minute modules. Two modules delivered during each of 4 weekly sessions. Sessions involved interactive exercises, games, brainstorming, roleplaying, videos, and group discussions.  Control: General health promotion, matched for group interaction and activity type. | Session 6: Correct condom use, discussed making condom use fun and pleasurable | 6 months  12 months | Unprotected vaginal intercourse (12 months)  ERR = 0·28**, 95% CI 0·08 – 0·97, p = 0·045  Frequency of condom use in the past 3 months  OR = 2·82**, 95% CI 1·18 – 6·74, p = 0·02  Self-efficacy for condom use  d = 0·32**, p = 0·04  HIV risk reduction knowledge  d = 0·79***, p < 0·001  Condom use knowledge  d = 0·31**, p = 0·04 |
| Hill & Abraham (2017)  UK  Wise up to condoms | RCT | Older teenagers in school between 16-18 years | 567 | Condom promotional leaflet, presented with quiz and prize draw  Control: No intervention given | Included information on how condoms can help to add fun to one’s sex life, without any loss of sensitivity or protection | 4 weeks | Attitudes (new partner), d = 0·46***, p < 0·001  Attitudes (steady partner), d = 0·30***, p < 0·01  Self-efficacy (prep), d = 0·71***, p < 0·001  Self-efficacy (use), d = 0·28**, p < 0·05  Condom use (new partner), d = -0·23, p > 0·05  Condom use (steady partner), d =0·12, p > 0·05 |
| Jemmott III et al. (2014)  South Africa  Men, Together Making  a Difference! | RCT | South African men who reported vaginal intercourse  Age range 18-45 | 1181 | Six 75-minute modules over 3 weeks delivered in highly structured, small groups.  Control: General health promotion, matched for group interaction and activity type | Video drama addressed effects of condom use on sexual enjoyment  Other activities addressed  the risk of different sexual behaviors, HIV’s  spread through a social network, condom-use  skills, making condoms fun and pleasurable,  and responding to partners’ concerns about using condoms. | 6 months,  12 months | Across 6- and 12-month assessments:  Consistent condom use in the last three months  OR = 1·32***, 95% CI 1·03 – 1·71, p = 0·008  Frequency condom use  OR = 1·41***, 95% CI 1·13 – 1·76, p = 0·002  Unprotected vaginal intercourse  OR = 0·95, 95% CI 0·81 – 1·13, p = 0·578 |
| Jemmott III et al. (2015)  USA  Being Responsible for Ourselves | RCT | African American men who have sex with men  Mean age 41.6 | 595 | HIV/STI risk reduction targeting condom use. Three, 90-min, one-on-one tailored sessions implemented over three weeks using standardized intervention manuals.  Control: Attention-matched general health promotion | Session 2 included part where participants considered ways to make condom use fun and pleasurable | 6 months  12 months | Across 6- and 12-month assessments:  Consistent condom use in the last three months  OR = 1·01, 95% CI 0·71 – 1·44, p = 0·95  Unprotected intercourse  OR = 0·99, 95% CI 0·69 – 1·42, p = 0·96 |
| Jemmott III et al., (2010)  USA  Be Proud! Be Responsible | Cluster RCT | African American adolescents between 13 – 18 years of age | 1707 | Six 50-minutes modules of interactive activities, films, small group discussions, experimental exercises, role-playing exercises, delivered in two sessions of 3 modules each. Intervention was implemented by community-based organizations. The intervention teaches that abstinence is the most effective way to prevent STDs, but it emphasizes that if adolescents do have sex they should use condoms.  Control: general health promotion intervention | Addresses attitudes toward condom use, skill and self-efficacy in using condoms, beliefs about negative consequences of condoms for sexual enjoyment, and skill and self-efficacy in negotiating condom use | 3 months  6 months  12 months | Over 12-month period:  Consistent condom use in last three months  OR = 1·39**, 95% CI 1·06 – 1·84, p = 0·02  Frequency of condom use  MD = 0·20**, 95% CI 0·02 – 0·39, p = 0·03  Condom use at last sexual intercourse  OR = 1·29*, 95% CI 1·00 – 1·67, p = 0·05 |
| Jemmott, Jemmott III, & O’Leary (2007)  USA  Sister-to-Sister: The Black Women’s Health Project | RCT | African American women in primary care settings  Mean age 27 | 564 | HIV/STD behavioral skill building: 20 minutes one-on-one (Arm 1) and 200 minutes in group (Arm 2).  HIV/STD information: 20 minutes one-on-one (Arm 3) and 200 minutes in group (Arm 4).  Control: General health promotion intervention | Arm 3: Group behavioural skills building involved addressing pleasure beliefs – specifically that condoms do not diminish enjoyment | 3 months  6 months  12 months | Skill group vs information groups:  3 months  Proportion protected sexual intercourse**, p = 0·02  Condom use at last intercourse, p = 0·92  Frequency of unprotected sexual intercourse**, p = 0·01  6 months  Proportion protected sexual intercourse*, p = 0·07  Condom use at last intercourse, p = 0·69  Frequency of unprotected sexual intercourse, p = 0·16  12 months  Proportion protected sexual intercourse*, p = 0·05  Condom use at last intercourse**, p = 0·01  Frequency of unprotected sexual intercourse**, p = 0·02 |
| Kerr et al. (2015)  USA  Focus on Youth | RCT | African American adolescents between 14 – 17 years of age | 1613 | Brief educational HIV-prevention curriculum and media campaign. 2 cities received culturally-tailored media intervention (TV and radio) promoting HIV reduction.  Control: Attention control | One of the media themes aimed to counteract perspectives regarding diminished pleasure associated with condom use and emphasize the condom’s benefits of reducing stress associated with risks of pregnancy and HIV | 3 months  6 months  12 months | Knowledge***, p < 0·0001 (3 months)  Knowledge***, p < 0·0001 (6 months)  Knowledge***, p < 0·01 (12 months)  Stigma*, p < 0·05 (3 months)  Stigma*, p < 0·05 (6 months)  Stigma*, p < 0·05 (12 months) |
| Lim et al. (2017)  Singapore | Quasiexperimental with pre- and post-tests and control group | Heterosexual men engaging in paid or casual sex  Age range 21-69 | 604 | Non-disease centric and non-stigmatizing intervention addressing sexual wellbeing through edutainment ‘talk shows’, an interactive web portal, and a public education/photo booth event. Additionally linkages to free, anonymous HIV testing as well as free access to ocndoms and lubricants.  Control: Distribution of education materials and provision of advice on healthy eating and physical activity | Entire intervention promotes sexual wellbeing. Edutainment component promotes positive attitudes towards sexuality, enhanced bonding, and sexual pleasure in marital and long-standing relationships.  Short 15- to  20-minute talk shows covered 6 themes, including tips on pleasurable sex with condom, information on masturbation | 6 months | Condom use at last vaginal sex (casual partner)  PR = 1·41, 95% CI 1·05 – 1·89  Condom use at last oral sex (casual partner)  PR = 1·70, 95% CI 1·11 – 2·61 |
| Marcell et al., (2013)  USA  Conservation Activities | Quasiexperimental with pre- and post-tests and control group | Young black males between 16-25 years | 197 | Three 60-minutes sessions led by two black male health educators, conducted immediately after GED class on consecutive days. Involves group discussions, interactive activities, presentations, demonstrations and hands on activities.  Control: No intervention | Session 2: Proper condom use, ways to make condom use more pleasurable | 3 months | STD knowledge  OR = 1·57***, 95% CI 1·44 – 1·71, p < 0·001  Attitude about sex enjoyment with condom  OR = 1·39, 95% CI 0·93 – 2·09, p > 0·05  Frequency of condom use  OR = 1·83***, 95% CI 1·24 – 2·71, p < 0·01  Used lube with condoms  OR = 23·61**, 95% CI 1·67 – 333·35, p < 0·05  Used condoms at last sex  OR = 1·10, 95% CI 0·88 – 1·34, p > 0·05 |
| Morrison-Beedy et al. (2005)  USA | RCT | Adolescent girls at Planned Parenthood between 15-19 years | 62 | Four, two-hour small group sessions aimed at HIV reduction, including role play and take-home activities  Control: Time and structure matched educational control | Behavioral skills components includes eroticizing safer sex | 3 months | Knowledge***, d = 0·63, p < 0·001  Behavioral skills**, d = 0·32, p = 0·04  Pros of condom use, d = 0·01, p = 0·93  Cons of condom use**, d = 0·35, p = 0·03  Vaginal sex with condom, d = 0·16, p = 0·50  Vaginal sex without condom, d = 0·26, p = 0·38 |
| Olley, Abbas, & Gidron (2011)*  Nigeria | Quasiexperimetnal with pre- and post-tests and control group | Women with HIV  Mean age 29.1 | 22 | Two 20 minute sessions of psychological inoculation, where participants were presented with barriers to condom use and discussed how to refute these  Control: 2, 20 minute sessions on safe sex health education | Addressed negative beliefs about condoms, such as that you cannot climax with condoms | 1 week | Pre- to post- results for intervention arm only:  Reduction in satisfaction barriers***, p=0·005  Self-efficacy for negotiating condom use**, p=0·03  Reduction in barriers for motivation**, p=0·048  Control vs intervention arm:  Always use condoms*, p=0·07 |
| Rosser et al. (2010)  USA  Men’s INTernet Study-II | RCT | Men who have sex with men, 18 years old and above | 650 | Internet based sexual health promotion intervention with multiple modules to build an overall personal ‘portrait of sexual health’. Intervention included cartoons, polls, FAQs, videos.  Control: Null control condition | Multiple modules provide positive and pleasure-inclusive views of sexuality, including a “hot sex” calculator, which  calculates the odds of great sex while demonstrating decision making in dating; a virtual  gym where men can explore body image concerns common in this population;, reflective journey exercises | 3 months  6 months  9 months  12 months | Unprotected anal intercourse (3 months)  IRR = 0·84*, 95% CI 0·70 – 1·01, p = 0·07  Unprotected anal intercourse (12 months)  IRR = 0·998, 95% CI 0·95 – 1·05, p = 0·94 |
| Sanderson & Yopyk (2007)  USA | RCT | College students  Mean age 19.6 | 220 | 2 condom promotion videotapes  Control: Waitlist | The core section of the two videos features a same-sex group of students discussing condom use (e.g. that it is appreciated when a partner suggests condom use, that using condoms shows respect for one’s partner, that it can be erotic when one’s partner suggests condom use) | Immediately after intervention  4 months | Proportion protected sex, p>0·05  Consistent condom use, p>0·05  Self-efficacy for suggesting condom use to a new partner  $\eta^{2}$ = 0·04**, p < 0·03  Self-efficacy for condom use, p>0·05  Self-efficacy for using a condom erotically, p>0·05 |
| Strathdee et al. (2013)  Mexico  Mujer mas segura | factorial RCT | Female sex workers who inject drugs  Mean age 33 | 584 | Four brief interactive single session conditions of about one-hour each, combining a (1) didactive or interactive sexual risk intervention to promote safer sex in the context of drug use, and (2) an interactive or didactic injection risk intervention to reduce sharing of needles/injection items  Control: didactic injection risk intervention and didactic sexual  risk intervention | The counsellor aimed to strengthen the woman’s commitment to using condoms by exploring ways to make condom use exciting and erotic | 12 months | Interactive Sex Risk intervetion (and Didactic Injection Risk Intervention) - Tijuana  HIV/STI incidence  IRR = 0·38**, 95% CI 0·16 – 0·89, p = 0·03  Interactive Sex Risk intervetion - Ciudad Juarez  HIV/STI incidence  IRR = 0·44*, 95% CI 0·19 – 0·99, p = 0·05 |
| Williams et al., (2012)*  USA  The Positive Choices Intervention | RCT | Heterosexual African American crack cocaine users living with HIV infection  Mean age 43.3 | 347 | Brief intervention composed of six, 60-minute mixed gender group sessions. Involves sharing, mapping, discussion.  Control: Standard intervention, group format sessions, predominantly informational | Session 2: exploring how to make sex with a condom pleasurable and fun  Session 4: Assessing personal sexual rights, sexual diversity, the need for intimacy, and positive sexual choices  Session 5: forum for examining gender role and relationship expectations, sexual scripts, and achieving sexual pleasure without vaginal sex | 3 months,  9 months | Time (3 and 9 months) per group interaction:  Condom use, p = 0·43  Intention, p = 0·27  Attitudes, p = 0·23  Partner norms, p = 0·24  Self-efficacy for performance*, p = 0·08  Situational self-efficacy**, p = 0·03  Self-efficacy for refusal, p = 0·28  Partner self-efficacy, p = 0·21  Self-efficacy for communication, p = 0·26 |
| Yancey et al. (2012)  USA  HIV-RAAP (HIV/AIDS Risk Reduction Among Heterosexually  Active African American Men and Women: A Risk Reduction Prevention Intervention) | RCT | Heterosexual African American men and women  Mean age 25.4 | 201 | 7 consecutive sessions of 2 hours each. Culture- and gender-sensitive coeducational curriculum, culturally congruent, in small groups, conducted at churches, YMCAs, community centers, etc.  Control: Standard HIV treatment and prevention written materials | Session 4: Condom Barrier Beliefs  Identify ways to achieve sexual satisfaction using safer sex methods | 7 weeks | Sexual behavior**, p = 0·02  Condom barrier beliefs*, p = 0·07  HIV risk knowledge***, p =0·006  Safer sex peer norms, p = 0·17  Conversations with sexual partners***, p =0·001 |
| Ybarra et al., (2018)  USA  Guy2Guy | RCT | Gay, bisexual, queer cisgender adolescent males between 14 – 18 years old  Mean age 16 | 302 | Multiple daily text messages over 5 weeks, with a 1 week booster delivered at 11 weeks. Content discussed HIV information, motivation, behavioral skills and included interactive features like a text buddy  Control: Attention-matched general health text messaging program | Behavioral skills incorporated pleasure, such as how to use lubrication to reduce risk and increase pleasure.  Information on tips how to promote pleasurable safe sex. | 3 months | High HIV prevention motivation  OR: 2·56*, p = 0·05  High condom use motivation  OR: 1·12, p = 0·85 |

*Table 1.* Summary table of included studies. The participant characteristics and outcomes are reported as in the original texts without interpretation or conversion. An asterisk alongside the study citation indicates this study was included in the meta-analysis. The incorporation of pleasure was not mentioned in three published manuscripts. For Goldberg et al. (2009) pleasure elements are described in the intervention manual. Ybarra et al. (2018) and Strathdee et al. (2013) similarly do not mention pleasure in their manuscript but the corresponding publications detailing the intervention designs clearly state pleasure incorporation (Ybarra et al., 2017 and Vera et al. 2012, respectively).



Stars denote statistical significance, as reported in original publications, where *** p < 0·01, ** p < 0·05, * p < 0·1.
