# Supplementary material for: What is the added value of incorporating pleasure in sexual health interventions? A systematic review and meta-analysis
Source: PLoS One. 2022 Feb 11;17(2):e0261034. doi: 10.1371/journal.pone.0261034 (PMC8836333; doi:10.1371/journal.pone.0261034)
Supplement: S1 Appendix — (DOCX) [file pone.0261034.s002.docx]

| **PubMed** | (("HIV Infections/prevention and control"[Mesh] OR "Pregnancy/prevention and control"[Mesh] OR "Sexually Transmitted Diseases/prevention and control"[Mesh] OR "Safe Sex/psychology"[Mesh] OR "sex education"[MeSH Terms] OR "contraception/education"[MeSH Terms] OR "contraception behavior"[MeSH Terms]) OR ((("HIV"[All Fields] OR "pregnancy"[All Fields] OR STI[All Fields] OR STD[All Fields]) n5 ("prevent*"[All Fields] OR "intervention"[All Fields])) OR (("reproductive health"[All Fields] OR "condom use"[All Fields] OR "safe sex"[All Fields]  OR "family planning"[All Fields] OR "contracepti*"[All Fields]) n5 ("promot*"[All Fields] OR "intervention"[All Fields])) OR "sex education"[All Fields] OR "sexual education"[All Fields])) AND (("matched controls"[All Fields] OR "natural controls"[All Fields] OR "quasiexperimental"[All Fields] OR  "control trial"[All Fields] OR "controlled trial"[All Fields] OR "controled trial"[All Fields] OR "RCT"[All Fields]) OR "Randomized Controlled Trial" [Publication Type]) |
| --- | --- |

**Appendix Table 1.** Search strategy as implemented in PubMed.
