# Supplementary material for: What is the added value of incorporating pleasure in sexual health interventions? A systematic review and meta-analysis
Source: PLoS One. 2022 Feb 11;17(2):e0261034. doi: 10.1371/journal.pone.0261034 (PMC8836333; doi:10.1371/journal.pone.0261034)
Supplement: S2 Appendix — (DOCX) [file pone.0261034.s003.docx]

| Pleasure spectrum | Manuscript description of intervention | Examples |
| --- | --- | --- |
| Red (Low pleasure inclusivity) | - Overall sex-negative, risk-reduction or disease-centredapproach - Includes one component that confirms that safer sex does not need to *stop* pleasure (e.g. dispels belief that condoms curtail pleasure) | “Three media themes emerged from these processes: (a) counteract perspectives regarding diminished pleasure associated with condom use and emphasize the condom’s benefits of reducing stress associated with the risk of pregnancy and HIV, (b) waiting to have sex indicates mutual respect for one’s partner, and (c) condoms should be used consistently.” (Kerr et al., 2015; p. 152)  “The group HIV/STD behavioral skill-building intervention was designed to increase skills regarding condom use and to allay participants’ concerns about the adverse effects of condom use on sexual enjoyment.” (Jemmott, Jemmott III, & O’Leary, 2007; p. 1035) |
| Amber (Medium pleasure inclusivity) | - Largely sex-negative or disease-centred approach - Includes at least one component that actively eroticizes condom or safer sex - May include limited focus on sex as potentially having benefits or present potential benefits in a “pleasure with a risk” context | “For example, men learned that condoms come in a variety of sizes and shapes, and they learned about the value of periodically adding water-based lubricants to condoms during sexual intercourse. Men learned, by demonstration from the lay health adviser, that oil-based lubricants can quickly erode latex condoms. Enhancing men’s motivation to use condoms was an integral component of the session. Throughout the session, the advisor encouraged men to feel good about using condoms, to experience condoms as being compatible with sexual pleasure, and to actively protect themselves from future STD acquisition. The lay health adviser constantly attempted to equate condom use with an investment in the men’s future.” (Crosby et al., 2009; p. S98)  “The core components of this intervention focus on both sexual and drug risk reduction (1) encouraging both partners to disclose and identify mutual drug-related and sexual risks; (2) modeling, role-playing and practicing couple communication, negotiation and problem solving skills that both partners may employ together to reduce their drug-related and sexual risks; (3) practicing technical condom use placement skills along with a broader repertoire of pleasurable safer sex activities and syringe disinfection skills; and (4) enhancing the couple’s motivation to protect each other and set mutual risk-reduction goals. Facilitators were trained to validate the relationship’s strengths of commitment, love, trust, and empower the dyad to enact protective behaviors.” (El-Bassel et al., 2018; p. 4) |
| Green (High pleasure inclusivity) | - Overall sex positive approach - Non-disease centric, overall approach does not stigmatize sex - Pleasure and sex positivity are the basis for the intervention or most of its components - Considers desire, sexuality, intimate relationships as a normal need and want in life | “The PEAS [Programa de Educação Afetivo-Sexual] Belgo program was based on the principle that sex education is a right, and an essential component of the personal and social development of adolescents. The program was inserted within the context of sexual and reproductive rights, and dealt not only with the risks involved in unsafe sexual practices but also focused on the positive aspects of sexuality. It emphasized the importance of intra and interpersonal relationships in the context of a healthy and pleasurable sex life, and stimulated gender equity with the aim of reducing the vulnerability of adolescents.” (Andrade et al., 2009; p. 4)  “The intervention program was nondisease centric and non stigmatizing addressing sexual well-being, promotion of condom use and HIV/STI testing using behavioral, biomedical and structural strategies. It focused on casual rather than commercial sex to reduce stigma. This also drives away the fear and threat of prosecution because commercial sex is illegal in EEs [entertainment establishments] in Singapore. Behavioral and educational strategies included edutainment, interactive Web-based portal and public education event/photo booth. Edutainment activities aimed to promote positive attitudes toward sexuality and enhance bonding and sexual pleasure in marital/long-standing relationships.” (Lim et al., 2017; p. 541)  ‘It [the intervention] eroticizes safer sexand aims to create a safe space in which black women can connect with their sexuality in ways that are positive and  self-loving rather than shameful or degrading.’ [Diallo, 2010; p519] |

**Appendix Table 2**. This table illustrates the diversity of ways pleasure is described within the peer-reviewed literature included in this review. We do not propose categorical cut-offs but instead consider these a spectrum ranging from “Red” – “Amber” – “Green”.
