# Supplementary material for: What is the added value of incorporating pleasure in sexual health interventions? A systematic review and meta-analysis
Source: PLoS One. 2022 Feb 11;17(2):e0261034. doi: 10.1371/journal.pone.0261034 (PMC8836333; doi:10.1371/journal.pone.0261034)
Supplement: S3 Appendix — (DOCX) [file pone.0261034.s004.docx]

| **Study** | **Bias due to randomization** | **Bias due to deviations from intended interventions** | **Bias due to missing outcome** | **Bias due to outcome measurement** | **Bias due to outcome selection** |
| --- | --- | --- | --- | --- | --- |
| **Andrade et al., (2009)** | High | Some concerns | Some concerns | Some concerns | Some concerns |
| **Bauermeister et al. (2019)** | Low | Low | Low | Low | Low |
| **Brown et al. (2019)** | Some concerns | Low | Low | Low | Low |
| **Champion & Collins (2011)** | Some concerns | Some concerns | Low | Low | Low |
| **Coleman et al. (2009)** | Low | Some concerns | Low | Some concerns | Low |
| **Copenhaver, Lee, Baldwin (2013)** | Low | Some concerns | Low | Low | Low |
| **Crosby et al. (2014)** | Low | Low | Low | Some concerns | Low |
| **Crosby et al. (2018)** | Low | Some concerns | High | Some concerns | Low |
| **Crosby et al., (2009)** | Some concerns | Low | Low | Low | Low |
| **Cruess et al. (2018)** | Low | Low | Low | Low | Low |
| **Diallo et al. (2010)** | Low | Low | Low | Low | Low |
| **El-Bassel et al., (2011)** | Low | Low | Low | Low | Some concerns |
| **Ferrer et al. (2011)** | Low | Low | Some concerns | Some concerns | Low |
| **Garcia-Vazquez, Quinto, Agullo-Tomas (2019)** | High | Some concerns | Some concerns | Some concerns | Some concerns |
| **Goldberg et al. (2009)** | Low | Low | Low | Low | Low |
| **Gollub et al. (2010)** | Low | Low | Low | Low | Low |
| **Heeren et al. (2013)** | Low | Low | Low | Low | Low |
| **Hill & Abraham (2017)** | Low | Low | Low | Low | Low |
| **Jemmott III et al. (2014)** | Low | Low | Low | Low | Low |
| **Jemmott III et al. (2015)** | Low | Low | Low | Low | Low |
| **Jemmott III et al., (2010)** | Low | Low | Low | Low | Low |
| **Jemmott, Jemmott III, & O’Leary (2007)** | Low | Low | Low | Low | Low |
| **Kerr et al. (2015)** | Some concerns | Some concerns | Some concerns | Low | Low |
| **Lim et al. (2017)** | High | Some concerns | Some concerns | Some concerns | Low |
| **Marcell et al., (2013)** | High | Low | Some concerns | Some concerns | Low |
| **Morrison-Beedy et al. (2005)** | Some concerns | Some concerns | Low | Some concerns | Low |
| **Olley, Abbas, & Gidron (2011)** | Low | Low | Low | Some concerns | Low |
| **Rosser et al. (2010)** | Low | Low | Low | Some concerns | Low |
| **Sanderson & Yopyk (2007)** | Some concerns | Some concerns | Low | Low | Low |
| **Strathdee et al. (2013)** | Low | Low | Low | Low | Low |
| **Williams et al., (2012)** | Low | Low | Low | Low | Low |
| **Yancey et al. (2012)** | Some concerns | Some concerns | Low | Some concerns | Some concerns |
| **Ybarra et al., (2018)** | Low | Low | Low | Low | Low |

**Appendix Table 3.** Quality assessment ratings. We used the Cochrane Collaboration’s Risk of Bias 2 (RoB2) tool for RCTs and cluster RCTs. The RoB2 provides ratings ranging from Low risk – Some concerns – High risk (coded above as Low – Some concerns – High) for different sources of potential risk. These are bias due to the randomization process, deviations from intended interventions, missing outcome data, measurement of outcomes, and selection of reported results. For cluster RCTs there is an additional category, bias from recruitment of participants. For the only cluster RCT in our sample, Jemmott III et al., (2010), bias from recruitment of participants was assessed as “Some concerns” from both reviewers.
