# Supplementary material for: What is the added value of incorporating pleasure in sexual health interventions? A systematic review and meta-analysis
Source: PLoS One. 2022 Feb 11;17(2):e0261034. doi: 10.1371/journal.pone.0261034 (PMC8836333; doi:10.1371/journal.pone.0261034)
Supplement: Attachment — Submitted filename: Response to Reviewers 1811 PONE ID D 21 23113.pdf [file pone.0261034.s006.pdf]

PONE-D-21-23113
What is the added value of incorporating pleasure in sexual health interventions? A systematic review and meta-analysis.
PLOS ONE

Dear Editor,

Thank you for the opportunity to submit a revised version of our manuscript. We are grateful for the productive and helpful comments from the Reviewers. We have taken their feedback into account and are submitting a revised manuscript accompanying this letter. Below we address the points raised from the Reviewers as well as journal requirements.

Reviewer #1: Strong/sound rationale and implications for the study. Well written. Nicely links with UN goals/larger conversations re health/sexual health. Good methodological approach re review. Good rigour with inclusion of meta analysis. Findings coalesce with focus on enhanced sex-positive messaging and inclusion of intimacy/pleasure etc as other relevant needs associated with sexual practices. Narrative synthesis of main findings from papers included in the review is well written. Discussion is sound.

Response 1: We thank Reviewer 1 for their encouraging comments and consider them a strong indication that our work will be of interest for the PLOS One readership.

Reviewer #2: Thank you for the opportunity to review this excellent paper. It is an interesting and important topic, concisely and clearly written. It uses sound methodology and results are explained clearly with an appropriate level of detail included.
I have three very minor suggestions, which I would consider optional to revise:
1. One area could be added to in the Discussion – the lack of inclusion of pleasure in sexuality education is probably not due to its effectiveness, but more about political and moral judgements. This could be discussed further.
2. This might be an issue with the format of the proof, but I can't see any titles/legends on the figures.
3. It would be helpful if the 8 studies included in the meta-analysis were identified in the table somehow so they can be easily found.

Response 2: We thank Reviewer 2 for their productive comments. We have considered and agreed with all three minor suggestions.

1. We agree that political and moral judgements could likely underpin, at least in part, the lack of inclusion of pleasure in sexuality education. In fact, we would agree with a stronger

version of the Reviewer's suggestion as well -- namely that such moral and political contexts likely affect the implementation of interventions that incorporate pleasure in broader contexts, outside of education as well. We have now addressed this point in the Discussion (p. 10) and have added a reference to a recent paper that discusses these issues in the context of challenges for carrying out systematic reviews on this topic (Philpott et al., 2021):

In terms of limitations, it is possible that we may have missed interventions incorporating pleasure because their write-ups did not indicate pleasure was a part of the intervention's components or delivery. The way interventions are described is often guided by considerations linked to funding, publication, cultural and other biases. *We acknowledge that such contextual and financial factors may also limit which interventions can be implemented in the first place. We discuss these issues as they relate to challenges for carrying out a systematic review and understanding effectiveness elsewhere*[55]*. In so far as how intervention description may affect inclusion, we have tried to mitigate the potential impact of this as much as possible by contacting authors and reading intervention manuals.*

2. Thank you for bringing our attention to this. We have tried to provide all relevant figures in pdf in order for high resolution to be available. We provide the table and figure titles and legends at the end of our manuscript and provide a copy here.

   Figures and tables in text.
   Figure 1. Flowchart depicting study selection.
   Figure 2. Forest plot.
   Figure 3. a) Funnel plot. b) Funnel plot following trim and fill procedure.
   Table 1. Study characteristics.

   Appendix.
   Appendix Table 1. Search strategy as implemented in PubMed.
   Appendix Table 2. Assessment of the different ways pleasure is described in articles.
   Appendix Table 3. Quality assessments based on the Cochrane Collaboration's RoB2 tool.

3. It would be helpful if the 8 studies included in the meta-analysis were identified in the table somehow so they can be easily found.

We have now done so by adding an asterisk next to the short study citation, such as for instance: Andrade et al. (2009)*.
We have clarified this by adding the following in the Table 1 legend: *An asterisk alongside the study citation indicates this study was included in the meta-analysis.*

Journal Requirements:

1. Please review your reference list to ensure that it is complete and correct. If you have cited papers that have been retracted, please include the rationale for doing so in the manuscript text, or remove these references and replace them with relevant current references. Any changes to the reference list should be mentioned in the rebuttal letter that accompanies your revised manuscript. If you need to cite a retracted article, indicate the article's retracted status in the References list and also include a citation and full reference for the retraction notice.

We have checked this directly in Zotero and at the time of writing believe the reference list is complete and correct, and that no retracted papers are cited. We have added one new citation as described in Response 2, Point 1.

Response 3:

2. While revising your submission, please upload your figure files to the Preflight Analysis and Conversion Engine (PACE) digital diagnostic tool, https://pacev2.apexcovantage.com/. PACE helps ensure that figures meet PLOS requirements. To use PACE, you must first register as a user. Registration is free. Then, login and navigate to the UPLOAD tab, where you will find detailed instructions on how to use the tool. If you encounter any issues or have any questions when using PACE, please email PLOS at figures@plos.org. Please note that Supporting Information files do not need this step.

After using PACE, we now provide .tif versions of our Figures 1, 2, 3 (a and b). We remain available for any formatting or other required changes.

We have also taken the liberty of changing the reference to the upcoming Generation Equality Forum on page 3 as this is now past.

Thank you once more to both Reviewers for the insightful comments.

Sincerely,

Anne Philpott
